# Platform algorithmic stress and career commitment of Chinese gig workers: a double-edged sword effect study

**DOI:** 10.3389/fpubh.2026.1824610

**Published:** 2026-06-01

**Authors:** Lexuan Xu

**Affiliations:** College of Information and Electrical Engineering, China Agricultural University, Beijing, China

**Keywords:** algorithmic stress, career commitment, job crafting, relative deprivation, gig worker

## Abstract

The rise of the platform economy has not only created numerous gig employment opportunities but also subjects gig workers to algorithmic stress. While existing research predominantly focuses on the negative consequences of algorithmic stress, many gig workers still exhibit high career commitment. To explain this paradox, this study draws on Self-Determination Theory and surveys 354 full-time Chinese gig workers, proposing a dual-path mediation model. Results reveal two opposing indirect effects of algorithmic stress on gig workers' career commitment. Firstly, algorithmic stress has a positive impact on gig workers' job crafting behaviors, which in turn enhance their career commitment. Secondly, algorithmic stress also has a on gig workers' sense of relative deprivation, which subsequently reduces their career commitment. This study advances algorithmic stress research by uncovering both facilitative and inhibitive pathways, extends Self-Determination Theory to algorithmic management, and offers practical insights for platform algorithm design so as to promote high-quality gig employment.

## 1 Introduction

With the rapid development of technological revolution and industrial transformation, the widespread application of information technologies (e.g., the mobile Internet, big data, and artificial intelligence) has profoundly reshaped the socioeconomic landscape. This has given rise to gig economy, covering many industries such as catering, retail, logistics, housekeeping, education. The review defined the gig economy as “people using apps [also commonly known as platforms] to sell their labor under flexible, contract-free arrangements” (1, 2). Gig work is usually typified by four characteristics: irregular work schedules; workers providing some or all capital (e.g., mobile phones, cars, or bikes); piece-rate work remuneration; and work being arranged and/or facilitated by digital platforms (3). Gig workers primarily comprise online delivery riders, ride-hailing drivers, and internet marketers, who have become an important workforce in China. In 2023, the results of the Ninth National Survey of the Workforce in China indicated that the total number of gig workers was 84 million, which accounted for 21% of the total workforce. And the majority of them are young workers aged below 35. Such new employment forms provide new career choices for many workers, create lots of opportunities for low-income groups, the unemployed and rural migrants. Gig workers have played a vital role in social harmony and stability.

Although gig economy has created more job opportunities, it has also introduced a novel form of labor control. Through algorithmic systems, platforms exercise precise control over gig workers' labor processes, performance, and income, forming an intangible “digital cage.” For gig workers, algorithms act as invisible “managers,” controlling their entire labor process and outcomes via automatic dispatch, route planning, ratings, and reward-punishment mechanisms (4). Algorithmic control brings gig workers an unprecedented experience of work stress, characterized by a high degree of opacity, intensified surveillance, and constant uncertainty (5). Work stress under differs fundamentally from that experienced by employees in traditional organizations. First, in traditional organizations, employees' work stress typically arises from managers' workplace inspections and direct oversight (6, 7). But for gig workers, the stressor originates from invisible, data-driven algorithmic management. Second, employee stress in traditional organizations typically occurs at a specific point or stage, whereas gig work appears to have no clear boundaries. Algorithmic stress covers the entire labor process, from pre-work to on-the-job to post-work (8). Third, since gig workers are subject to invisible platform management and must interact with customers, their resulting stress is potentially more diverse and complex (9).

### 1.1 Literature review

Overall, current research on algorithmic stress at the individual-level remains insufficient (9). Most existing studies focus on the negative work experiences algorithmic pressure brings to gig workers, such as perceived lower social status, increased workload, emotional exhaustion, reduced proactive service behavior, increased work withdrawal, decreased service performance, and heightened turnover intention (8–10). These studies often call for platforms to find ways to prevent or mitigate algorithmic stress and related strain responses. This suggests that this unique stress experience under algorithmic control negatively impacts gig workers' work attitudes and outcomes. For many people, gig work is perceived not as a lifelong career but as a transient bridge or income supplement, reflecting a low willingness for long-term engagement (11). Consequently, generally low career commitment would not be surprising.

However, despite being immersed in this algorithmically constructed stressful context, a number of gig workers show sustained career dedication and retention intention. In September 2025, a research group from the New Employment Forms Research Center in China, based on a questionnaire survey of over 5,400 gig workers, found that the willingness to continue working among ride-hailing drivers was 75.7%, and the retention intention among food delivery riders was 68.4%. Thus, platform algorithmic stress does not exert a purely inhibitory effect on gig workers' career commitment. That is, stressors are not always deleterious (7). Under algorithmic stress, many gig workers still identify with their occupation and maintain sustained involvement. For platform enterprises, the workforce is a core resource, and those long-tenured, continuously invested gig workers with high commitment are particularly valuable. High levels of career commitment help improve service processes among gig workers, thereby enhancing customer satisfaction and promoting the sustainable development of platform enterprises. However, current research on career commitment has primarily focused on employees in traditional organizations (12), teachers (13), and civil servants (14). In contrast, relatively little attention has been paid to the career commitment of the emerging workforce employed by online labor platforms (1, 15). Given the large and rapidly growing number of gig workers, their identification with and commitment to gig work are crucial for employment stability, social harmony, and economic development. Therefore, we believe it is highly necessary to conduct an in-depth investigation into the influencing factors of gig workers' career resilience.

### 1.2 Theoretical framework and research questions

Why do identical algorithmic stressors lead to such different career commitment responses among gig workers? We propose that algorithmic pressure produces a double-edged sword effect on career commitment: on one hand, it may strengthen commitment through a positive mediating mechanism; on the other hand, it may weaken commitment through a negative mediating mechanism. The two pathways operate in parallel and compete to determine the net effect. A comprehensive inquiry into this issue serves not only to enhance platform competitiveness but also to improve gig workers' employment quality. Therefore, this paper aims to explore the mediating mechanisms through which platform algorithmic pressure translates into gig workers' career commitment, seeking to unlock this “black box” and provide a new theoretical perspective for understanding new labor relations in the digital age.

Self-Determination Theory (SDT) provides an explanatory framework for understanding this issue. SDT emphasizes that the satisfaction of basic psychological needs (autonomy, competence, and relatedness) can effectively stimulate intrinsic motivation, thereby promoting positive psychological states and behaviors (16). According to SDT, when individuals face external pressure, they may regain a sense of control, efficacy, and interpersonal connection at work through autonomous behaviors (such as job crafting), transforming pressure into an opportunity to facilitate psychological need satisfaction (17). This study posits that even under the stressful environment of algorithmic control, when individuals appraise external pressure as a “challenge stressor,” gig workers may believe this pressure can satisfy their innate psychological needs, thereby eliciting positive emotions and psychological responses (18). Consequently, they will proactively adopt a series of strategic adaptive behaviors for job crafting: including redefining task boundaries, reconstruct work meaning cognition, and establishing new cooperative networks (19). These job crafting behaviors not only help workers achieve compensatory satisfaction of their psychological needs, but also effectively enhance their work engagement, thereby fostering identification with their work and roles (20). Thus, workers can construct a positive job identity and form a sustained commitment to their gig occupation. Gig workers' career commitment stems from the reshaping of internal needs and work situations through proactive job adaptation under algorithmic pressure. Therefore, this study proposes job crafting plays a potential mediating effect between algorithmic stress and gig workers' career commitment.

On the other hand, Self-Determination Theory also provides a crucial perspective for understanding the negative cognitive-affective pathways that algorithmic stress may trigger. An individual's cognitive appraisal of stress significantly influences the degree of psychological need satisfaction, subsequently affecting their intrinsic motivation and long-term behavioral tendencies (16). Specifically, when individuals appraise external pressure as a “hindrance stressor,” they perceive it as obstructing their growth, goal attainment, or psychological need fulfillment, thereby eliciting negative emotions and defensive psychological reactions (21). Gig workers may interpret algorithmic stress as a threat to their autonomy, competence, and belongingness if they perceive algorithmic management as standardized control, rigid time constraints, and an interpersonally alienating environment. Such perceptions can trigger relative deprivation, which is a negative cognitive-affective state rooted in social comparison and need frustration. Relative deprivation arises from the following three negative experiences: (1) a psychological discrepancy between “legitimate entitlements” and “actual circumstances;” (2) frustrated psychological needs; and (3) an inability to derive satisfaction from comparisons with others or with one's own expectations. Therefore, this study proposes relative deprivation as another key mediating mechanism through which algorithmic stress influences gig workers' career commitment.

Based on Self-Determination Theory, this study aims to examine the dual mediating roles of job crafting and relative deprivation in the process through which algorithmic stress stimulates gig workers' career commitment. On one hand, job crafting represents the positive adaptive path where gig workers proactively construct meaning and regain need satisfaction under algorithmic stress. On the other hand, relative deprivation reveals the negative psychological path gig workers may trap into after negative cognitive appraisal and persistent need frustration. Such two mediating paths constitute a “dual-path model” of algorithmic stress's impact on career commitment. One path leads toward psychological compensation and enhanced career commitment through proactive job crafting, and the other leads toward emotional alienation and diminished career commitment through relative deprivation. This research aims to provide a more comprehensive and dialectical understanding of the complex internal processes through which algorithmic management affects gig workers' occupational psychology.

## 2 Research hypotheses

### 2.1 Algorithmic stress and the dual cognitive appraisal

Job stress is a dynamic process in which employees exhibit a series of physiological, psychological, and behavioral responses when job demands exceed their coping capacity (22). With the rapid development of the gig economy, the number of gig workers has continuously increased, and their work stress has become a focal point for both academia and industry. Compared with traditional organizational employees, gig workers face greater income instability and job insecurity in platform work, along with higher level of new stressors such as work constraints, social isolation, and career uncertainty (9, 23, 24). These constitute the specific work pressure faced by the gig workforce, i.e., algorithmic stress. Existing research indicates that the algorithmic stress faced by gig workers includes three types: regulation stress, time stress, and alienation stress (10). In their research, regulation stress refers to the pressure gig workers experience during the online labor process due to automatic task allocation, real-time normative guidance, and tracking evaluation by the algorithmic platform. Time pressure refers to the sense of temporal urgency that gig workers experience during work due to real-time algorithmic tracking and regulation. It defines both the required tasks and the allotted completion time. Third, alienation stress refers to the perceived helplessness due to the lack of support resources from platforms, leaders and colleagues, leading to social relationship estrangement.

Folkman et al. (25) proposed that individuals would appraise whether and how stress affects their physical and mental wellbeing, including primary appraisal, secondary appraisal, and coping. In primary appraisal, the individual evaluates whether he or she has anything at stake in this encounter. For example: does this pose potential harm or benefit with respect to commitments, values, personal esteem, or goals? In secondary appraisal, the individual evaluates what, if anything, can be done to overcome or prevent harm or to improve the prospects for benefit. Based on this, Cavanaugh et al. (26) categorized work stress into two types: (a) challenge stressors and (b) hindrance stressors. Challenge stressors are work-related demands or circumstances that, although potentially stressful, have associated potential gains for individuals. Conversely, hindrance stressors are work-related demands or circumstances that tend to constrain or interfere with an individual's work achievement and that do not tend to be associated with potential gains for the individual. Coping is defined as the person's constantly changing cognitive and behavioral efforts to manage specific external and/or internal demands that are appraised as taxing or exceeding the person's resources (27). Research indicates that the appraisal process of stressors triggers specific emotional reactions and coping strategies, subsequently influencing behavior (28).

Given the distinct functional features of algorithms, algorithmic control may reshape the job attributes and occupational stressors faced by gig workers in novel and disruptive ways. On one hand, platform algorithms strictly regulate gig workers' labor process, offering real-time monitoring and constraint of any behavior that deviates from preset standards under the platform's objective functions (29, 30, 47). On the other hand, online labor platforms employ algorithms to continuously refine the labor process, deliver decision-relevant information to platform workers and facilitate multi-task coordination (29, 31). Given algorithmic control's dual technical attributes, many studies suggest that gig workers may simultaneously experience both challenge and hindrance appraisals of algorithmic stress (9). Gig workers' perceptions, cognitions, and appraisals of algorithmic control significantly influence their work attitudes and behaviors (32).

### 2.2 Algorithmic stress and job crafting

According to Self-Determination Theory, individuals can be proactive and engaged in their work, if the social conditions in which they are situated are autonomy supportive (49). For gig workers, they will proactively adapt to the influence of algorithmic control by reshaping job redesign. That is, algorithmic stress can promote gig workers' job crafting, enhancing the fitness between themselves and the platforms. Job crafting is a self-initiated, proactive behavior through which individuals adjust job demands and job resources according to job characteristics and individuals' capability and preference (19, 20). It can be categorized it into three types: task crafting, cognitive crafting, and relational crafting.

Specifically, gig workers transform algorithmic pressure into resources through the following job crafting behaviors: (1) Task crafting. When online platforms impose regulation and time stress. In response, gig workers may deploy counter-algorithmic strategies, such as multi-apping, optimizing order acceptance, planning work pace, and avoiding low-benefit orders. These strategies expand task control (47), which in turn enhances their skill variety, work autonomy, and intrinsic motivation. (2) Relational crafting. Online labor platforms create a work environment without human intervention. Such physical isolation deprives gig workers of interaction and support from colleagues and direct supervisors. To satisfy relation needs, gig workers may proactively build “virtual colleague networks” (e.g., WeChat-based peer groups), to form positive cooperative relationships with merchants or consumers. Thus, they can achieve social supports to alleviate relationship isolation, compensate for the lack of organizational identity (33). (3) Cognitive crafting. Under algorithmic pressure, gig workers' need for competence drives them to reconstruct the meaning of work, perceiving algorithmic rules as a “game” to be mastered rather than pure control. In summary, under algorithmic stress, the pursuit of satisfying autonomy, competence, and relational needs motivates gig workers to engage in job crafting. Therefore, this study proposes the following hypothesis:

H1: Algorithmic stress is positively related to gig workers' job crafting.

### 2.3 The mediating role of job crafting

Furthermore, research indicates that job crafting is a personalized and proactive form of job redesign (34) that helps employees alter their perceptions of job characteristics and construct a more positive sense of work meaning. It contributes to reducing physical discomfort and depressive symptoms while enhancing job satisfaction and commitment (35). In the algorithmic context, job crafting can help gig workers increase social resources, such as seeking guidance and support from peers, receiving positive feedback from merchants and consumers (4), thereby enhancing their sense of belonging. Additionally, job crafting can increase gig workers' structural resources, for example, by proactively learning new algorithmic knowledge, improving algorithmic literacy and work ability development, increasing autonomy in choosing work content and methods, satisfying challenging demands (e.g., proactively undertaking extra-role tasks), and enhancing their sense of work competence, while reducing hindering demands (e.g., avoiding difficult decisions or difficult customers) (34). In such circumstances, digital gig workers' enthusiasm, sense of belonging, and identification with platform work are likely to increase (36), consequently enhancing their career commitment.

H2: Job crafting positively influences gig workers' career commitment.
H3: Job crafting mediates the relationship between algorithmic stress and gig workers' career commitment.

### 2.4 Algorithmic stress and relative deprivation

According to Self-Determination Theory, individuals can also be passive and alienated in their work, if they experience social conditions as externally controlled (49). Unlike traditional organizations, algorithmic control embeds managerial authority deeply within the technical system, making control more opaque, normalized, and difficult to challenge. Gig workers seemly enjoy work autonomy, yet experience a strong sense of “deprivation,” which stems from a significant gap between their actual situation and perceived entitlements.

First, gig workers have autonomy over how they perform their work, but task allocation, pricing, and evaluation remain fully governed by algorithms. They cannot participate in rule-setting and lack effective channels to appeal against unfair decisions (37). Algorithms function as unchallengeable “rule enforcers,” thereby diminishing workers' sense of control over the labor process. Second, platforms leverage massive labor markets to enable real-time task matching. To maintain their income, workers must adapt to cross-time-zone and fragmented work rhythms. This often leads to sleep deprivation, disrupted daily routines, and social relationship alienation among gig workers (47). Therefore, under the facade of temporal autonomy, what actually takes place is a thorough encroachment of platform work upon the personal life domain. Third, in traditional employment relationships, colleagues, teams, and supervisors are important social support networks. They can provide emotional supports, skills guidance, and collective identity. However, the gig economy is eroding working conditions due to widespread designation of workers as independent contractors, as opposed to formal employees. In addition, platforms physically isolate daily interactions and collaboration with peers like leaders and colleagues (47). Labor scholars have also argued that these “flexible” employment arrangements result in precarity, with workers absorbing market risks and social responsibilities (48). When facing technical failures, customer disputes or health risks, gig workers are often isolated and helpless. Platforms as intermediaries often avoid employer responsibilities by shifting risks onto individuals, exacerbating workers' sense of non-belonging and helplessness. Therefore, what workers experience is not freedom, but a social deprivation of being abandoned by the platform system.

H4: Algorithmic stress positively influences gig workers' relative deprivation.

### 2.5 The mediating role of relative deprivation

In the workplace, when individuals can participate in work based on their own values and purposes, experience autonomy, and receive clear feedback and support at work, they are more likely to be motivated by autonomous motivation. In contrast, when workers are controlled or monitored by external forces, whether through rewards or power deterrence, although it may strongly stimulate specific controlled behaviors, workers typically do not internalize these behavioral rules and instead choose to reduce their effort scope (32, 38).

According to Self-Determination Theory, when gig workers appraise the external forces of control or surveillance intensified by algorithmic regulation pressure, time pressure, and alienation pressure as hindrance stressors, this constrains the satisfaction of their three basic psychological needs, i.e., autonomy, competence, and relatedness. Consequently, this weakens their intrinsic motivation, leading to cognitive comparisons during the labor process and inducing perceptions of personal disadvantage. With the continuous development of digital technology, the role of “manager” within organizations is gradually being replaced by digital technology. Algorithmic technological control may lead workers to experience alienation, as they may be deprived of the right to conceive of themselves as the directors of their own actions (39). The piece-rate system, order-grabbing mechanisms, panoramic monitoring, social ratings, and other rules established by platforms prevent gig workers from experiencing the “freedom” they had anticipated during the work process. Instead, they remain trapped in a disadvantaged position of information asymmetry, constantly enveloped in an atmosphere of anxiety and urgency. Ultimately, they become more “willing” to exert labor, seeking to prove their self-worth through speed (40). Within the existing platform management system, gig workers lack mechanisms for communications with the platforms, so they are in a disadvantaged position and cannot identify with the platforms.

As digital capital and its agents retreat backstage, platforms skillfully obscure labor-capital conflicts. In fact, what replaces this is gig workers' powerless complaints against the platform system's “algorithmic tyranny,” along with dissatisfaction and resentment toward consumers who give them negative reviews—i.e., labor-capital conflict. In other words, labor-capital contradictions subtly transform into human-machine contradictions, or horizontal conflicts between digital workers and clients, or even among digital workers competing with each other. Faced with an “aloof” platform system, digital workers with little bargaining power, having no better employment options, reluctantly resign themselves to becoming “digital serfs” (41). When a power imbalance exists between two or more parties, the party in the more disadvantaged position finds it difficult to defend one's rights, thereby generating negative emotions of relative deprivation (42). This sense of relative deprivation further transforms into organizational non-identification, workplace withdrawal behaviors, etc., thereby weakening gig workers' career commitment (11).

H5: Relative deprivation negatively influences gig workers' career commitment.
H6: Relative deprivation mediates the relationship between algorithmic stress and gig workers' career commitment.

**Figure 1:**
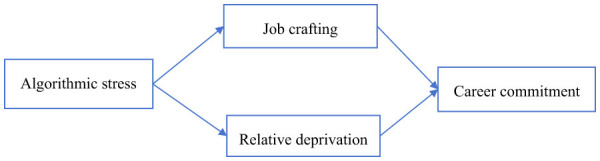
Theoretical model.

Based on the above hypotheses development, this paper constructed the following theoretical model (see Figure 1).

## 3 Research methods

### 3.1 Measurement

This study employed a questionnaire survey method. The scales used were adapted from established ones which have good reliability and validity in various researches. The questionnaire utilized a 5-point Likert scale, with options ranging from 1 (strongly disagree) to 5 (strongly agree); higher scores indicated a higher degree of agreement.

(1) Algorithmic stress. It was adapted from the scale developed by Gao et al. (10). This study selected the three dimensions: regulation stress, time stress, and alienation stress. Representative items include “The system constrains my behavior at any time, making me very nervous,” “Unexpected situations during order completion intensify my sense of time urgency,” and “I often feel lonely or helpless at work.” Cronbach's α was 0.953.
(2) Job crafting: It was developed by Slemp and Vella-Brodrick (43). The three dimensions are task crafting, cognitive crafting, and relational crafting. Representative items include “I learn new methods to improve my work,” “I know the importance of my work to the general public of consumers,” and “I participate in social activities related to this job.” Cronbach's α was 0.951.
(3) Relative deprivation: The scale by He and Chen (44) was used. Representative items include “Compared with peers around me, I feel my salary and benefits are relatively low,” “Compared with my own expectations or the past, I feel my salary and benefits are relatively low,” “Compared with the peers, the platform gives me fewer resources (status, reputation, rights, etc.),” and “Compared with my own expectations or the past, the platform gives me fewer resources (status, reputation, rights, etc.).” Cronbach's α was 0.901.
(4) Career commitment: Following the approach of (1), and based on the career commitment scale developed by Blau (45), 6 items were selected to measure gig workers' career commitment, such as “This is my ideal occupation,” “I like this occupation and don't want to give it up,” and “I don't want to change to another occupation.” Cronbach's α was 0.935.

Control variables. Statistical demographic characteristics were used as control variables, specifically including gender, age, education level, marital status, work tenure, daily working hours, and monthly income. To ensure research validity, the study sample was specifically defined to “full-time” gig workers. Unlike part-time gig workers who engage in gig work as supplemental income, full-time gig workers consider it their primary occupation and source of income. Their dependence on gig work is higher, their investment deeper, and issues of professional identity are more pronounced. Therefore, selecting this group allows for a purer revelation of the genuine formation mechanisms of career commitment within this novel, de-organizationalized occupational form, avoiding dilution of the core research question by the temporary nature and low investment characteristics of part-time workers.

### 3.2 Data collection

This study used a questionnaire survey method. The target respondents were primarily food delivery riders, couriers, and ride-hailing drivers from various regions. The survey was conducted from January 18 to 26, 2026 on the questionnaire platform Credamo. To motivate respondents to complete the questionnaire carefully and ensure the validity of the online questionnaires, respondents were given certain monetary rewards based on the content and duration of their completion. A total of 400 questionnaires were distributed, and 354 valid questionnaires were ultimately collected, yielding a valid response rate of 88.5%. This study used SPSS 24.0 to analyze the characteristics of the samples, with results shown in Table 1. Among the 354 samples, 261 were male (73.7%), 260 were aged 30 or above (74.4%), 203 had an education level of junior college or below (57.3%), 265 were married (74.9%), 227 had been engaged in their current occupation for over 1 year (64.1%), 297 worked more than 8 h per day (83.9%), and 208 had a monthly income above 5,000 RMB (58.8%).

**Table 1:** Descriptive statistics (*N* = 354).

Variable	Category	Frequency	Percentage (%)
Gender	Male	261	73.7
	Female	93	26.3
Age	20–29	94	26.6
	30–39	116	32.8
	40–49	111	31.4
	50 and above	33	9.3
Education level	Middle school	62	17.5
	High school	141	39.8
	Junior college	123	34.7
	Bachelor's degree	28	7.9
Marital status	Other	3	0.8
	Unmarried	86	24.3
	Married	265	74.9
Work tenure	6 months and below	45	12.7
	6 months−1 year (incl. 1 year)	82	23.2
	1–2 years (incl. 2 years)	115	32.5
	Over 2 years	112	31.6
Daily working hours	4–8 h	57	16.1
	8 h and above	297	83.9
Monthly income (RMB)	Below 3,000	11	3.1
	3,000–5,000	135	38.1
	5,000–8,000	154	43.5
	Above 8,000	54	15.3

## 4 Data analysis and hypothesis test

### 4.1 Confirmatory factor analysis and common method bias test

First, to test the discriminant validity of the variables, this study used AMOS 21.0 software to conduct confirmatory factor analysis (CFA) on algorithmic stress, job crafting, relative deprivation, and career commitment (as shown in Table 2). The results indicated that the four-factor model demonstrated a good fit (χ^2^/*df* = 2.919, CFI = 0.896, TLI = 0.888, SRMR = 0.051, RMSEA = 0.074), which was significantly better than other alternative competitive models, suggesting good discriminant validity among the variables. Second, a common method bias test was conducted. Using Harman's single-factor test, the results showed that no single factor emerged, and the first principal component accounted for 35.189% of the variance, which is below the critical threshold of 40%. Therefore, common method bias is not a serious concern in this study.

**Table 2:** Results of confirmatory factor analysis.

Model	*χ^2^*	*df*	*χ^2^*/*df*	CFI	TLI	SRMR	RMSEA
Four-factor	1,617.204	554	2.919	0.896	0.888	0.051	0.074
Three-factor	4,122.490	557	7.401	0.651	0.627	0.169	0.135
Two-factor	5,071.293	559	9.072	0.558	0.530	0.190	0.151
Single-factor	6,289.237	560	11.231	0.439	0.404	0.205	0.170

Four-factor: Algorithmic stress, Job crafting, Relative deprivation, Career commitment; Three-factor: Algorithmic stress + Job crafting, Relative deprivation, Career commitment; Two-factor: Algorithmic stress + Job crafting + Relative deprivation, Career commitment; Single-factor: Algorithmic stress + Job crafting + Relative deprivation + Career commitment.

### 4.2 Correlation analysis and reliability test

Table 3 presents the correlation coefficients and reliability test results for the study variables. As shown in Table 3, all Cronbach's α coefficients of the variables exceed the critical value of 0.7. Algorithmic stress is positively correlated with job crafting (*r* = 0.363, *p* < 0.01); job crafting is positively correlated with career commitment (*r* = 0.540, *p* < 0.01); algorithmic stress is positively correlated with relative deprivation (*r* = 0.348, *p* < 0.01); and relative deprivation is negatively correlated with career commitment (*r* = −0.389, *p* < 0.01). These results provide initial evidence for the following research hypotheses.

**Table 3:** Descriptive statistics and correlation analysis.

Variable	Gender	Age	Education level	Marital status	Tenure	Daily working hours	Monthly income	Algorithmic stress	Job crafting	Relative deprivation	Career commitment
Gender	-										
Age	0.117^*^	-									
Education level	−0.096	−0.127^*^	-								
Marital status	0.058	0.121^*^	−0.069	-							
Tenure	−0.046	0.118^*^	0.029	0.179^**^	-						
Daily working hours	−0.035	0.027	−0.010	0.104	0.078	-					
Monthly income	0.060	0.095	−0.009	0.108^*^	0.046	0.146^**^	-				
Algorithmic stress	0.052	−0.042	0.015	−0.020	−0.085	−0.001	−0.007	(0.953)			
Job crafting	0.098	0.016	0.037	0.039	−0.025	0.126^*^	0.067	0.363^**^	(0.951)		
Relative deprivation	0.008	−0.01	−0.022	−0.177^**^	−0.151^**^	−0.178^**^	−0.094	0.348^**^	−0.159^**^	(0.901)	
Career commitment	0.007	−0.012	0.098	0.071	−0.044	0.079	0.049	0.038	0.540^**^	−0.389^**^	(0.935)
M	1.263	3.235	2.331	1.740	2.831	2.839	2.709	3.549	4.119	2.430	3.621
SD	0.441	0.948	0.855	0.458	1.015	0.368	0.758	1.098	0.711	1.026	1.026

^*^p < 0.05; ^**^p < 0.01.

Values in parentheses on the diagonal represent the reliability coefficients for each variable. M denotes mean, and SD denotes standard deviation.

### 4.3 Hypothesis testing

(1) Test of direct effects. To test the four direct effects (H1, H2, H4, and H5), this study adopted SPSS 21.0 to conduct hierarchical regression analysis. As shown in Table 4, algorithmic stress has a significant positive effect on gig workers' job crafting (M2: β = 0.233, *p* < 0.001), supporting Hypothesis H1. Job crafting has a significant positive effect on gig workers' career commitment (M5: β = 0.774, *p* < 0.001), supporting Hypothesis H2. Algorithmic stress has a significant positive effect on relative deprivation (M7: β = 0.318, *p* < 0.001), supporting Hypothesis H4. Relative deprivation has a significant negative effect on gig workers' career commitment (M8: β = −0.396, *p* < 0.001), supporting Hypothesis H5.

**Table 4:** Direct effect analysis.

Control variable	Job crafting		Career commitment			Relative deprivation		Career commitment
	M1	M2	M3	M4	M5	M6	M7	M8
Gender	0.164	0.132	0.025	0.021	−0.102	0.008	−0.036	0.028
Age	0.004	0.014	−0.007	−0.005	−0.010	0.028	0.041	0.004
Education level	0.043	0.038	0.128^*^	0.127^*^	0.095	−0.032	−0.040	0.115
Marital status	0.039	0.041	0.177	0.178	0.147	−0.316^**^	−0.313^**^	0.052
Tenure	−0.027	−0.007	−0.068	−0.066	−0.047	−0.115^*^	−0.088	−0.114^*^
Daily working hours	0.240^*^	0.233^*^	0.203	0.202	0.017	−0.411^**^	−0.420^**^	0.040
Monthly income	0.039	0.040	0.046	0.046	0.016	−0.075	−0.073	0.017
Depend variable								
Algorithmic stress		0.233^***^		0.030			0.318^***^	
Mediating variable								
Job crafting					0.774^***^			
Relative deprivation								−0.396^***^
*F*	1.649	8.208^***^	1.355	1.230	18.956^***^	3.947^***^	10.009^***^	8.977^***^
*R^2^*	0.032	0.16	0.027	0.028	0.305	0.074	0.188	0.172
ΔR^2^	0.013	0.140^***^	0.007	0.005	0.289^***^	0.055^***^	0.170^***^	0.153^***^

^*^p < 0.05; ^**^p < 0.01; ^***^p < 0.001.

(2) Test of mediation effects. This study uses a Bootstrap resampling method with 5,000 iterations to examine the mediating roles of job crafting and relative deprivation. The analysis results, shown in Table 5, indicate that the indirect effect of algorithmic stress on career commitment via job crafting is 0.2029, with a 95% confidence interval of [0.1233, 0.2897], excluding 0. This demonstrates a significant mediating effect of job crafting, supporting Hypothesis H3. Similarly, the indirect effect of algorithmic stress on career commitment via relative deprivation is −0.1481, with a 95% confidence interval of [−0.1908, −0.1077], excluding 0. This demonstrates a significant mediating effect of relative deprivation, supporting Hypothesis H6.

**Table 5:** Mediation effect test for job crafting and relative deprivation.

Mediation path	Indirect effect coefficient	SE	Boot 95% CI	
			LLCI	ULCI
Algorithmic stress → Job crafting → Career commitment	0.2029	0.0423	0.1233	0.2897
Algorithmic stress → Relative deprivation → Career commitment	−0.1481	0.0213	−0.1908	−0.1077

## 5 Research conclusions and discussion

### 5.1 Research conclusions

Based on Self-Determination Theory, this study constructed and empirically tested the dual-path mechanism through which algorithmic stress influences career commitment, using survey data from 354 Chinese gig workers. The main conclusions are as follows. First, algorithmic stress has a significant positive impact on gig workers' job crafting, and job crafting has a significant positive impact on their career commitment. Furthermore, job crafting significantly mediates the relationship between algorithmic stress and career commitment. This indicates that algorithmic stress can trigger gig workers' challenge appraisal, prompting them to engage in proactive behaviors such as task crafting, relational crafting, and cognitive crafting. Through these actions, they regain a sense of control, efficacy, and social connection in a constrained environment, thereby compensatorily satisfying their basic psychological needs for autonomy, competence, and relatedness (17). Such path of “algorithmic stress → proactive crafting → need satisfaction → enhanced commitment” confirms that, under specific conditions, algorithmic stress can act as a catalyst driving individual positive adaptation and the construction of professional identity. Hypotheses H1, H2, and H3 are all supported by the data.

Second, algorithmic stress has a significant positive impact on gig workers' relative deprivation, and relative deprivation has a significant negative impact on their career commitment. Moreover, relative deprivation significantly mediates the relationship between algorithmic stress and career commitment. This suggests that when gig workers appraise algorithmic stress as a hindrance stressor, they perceive it as structurally suppressing their psychological needs, generating a strong sense of discrepancy between “deserved entitlements” and “actual situation” (11). This relative deprivation weakens their intrinsic motivation and professional identity, subsequently leading to diminished career commitment. This path of “stress → hindrance appraisal → relative deprivation → diminished commitment” reveals a potentially negative psychological cycle induced by algorithmic management. Hypotheses H4, H5, and H6 are all supported by the data.

In summary, the ultimate impact of algorithmic stress on gig workers' career commitment results from the combined forces of the positive facilitative path of “job crafting” and the negative inhibitive path of “relative deprivation.” Workers' cognitive appraisal of stress serves as the key cognitive switch determining which path they enter. This explains the seemingly contradictory phenomenon observed in platform surveys, where relatively high retention intentions coexist with identity dilemmas. The research conclusions indicate that enhancing gig workers' career commitment cannot solely rely on individual resilience; it is more crucial to optimize algorithm design at the source, reducing its “hindrance” characteristics and creating space for workers' proactive adaptation (8).

### 5.2 Theoretical contributions

First, existing research on algorithmic stress is still in its early stages. Most researches focus on concept definition, measurement tool development, and preliminary correlational findings (e.g., algorithmic stress leading to exhaustion and work withdrawal) (10). There is a general lack of in-depth exploration of its mediating mechanisms, leaving research conclusions at the descriptive level of “what it is” without systematically answering the “why” and “how” questions. This study advances the outcome research on algorithmic stress from simple causal association to dual mechanism explanation, opening the “black box” of how algorithmic stress affects individual psychology and behavior. It promotes the theorization of algorithmic stress research and significantly deepens the academic understanding of this emerging construct.

Second, the research perspective shifts from “platform commitment” to “career commitment,” expanding a new dimension in the study of gig workers' career sustainability. Existing research often focuses on gig workers' commitment to specific platforms or behaviors like online working hours (36, 46), yet neglects workers' long-term identification with and willingness to invest in the occupation itself (i.e., career commitment). This study places career commitment, a classic organizational behavior construct, within the gig context for examination, responding to gig workers' deep-seated aspiration for “a real profession.” It elevates the research focus to the more profound and essential level of valuing one's professional identity.

Third, this study proposes a “dual-path model” of algorithmic stress's impact on career commitment, deepening and extending the application of Self-Determination Theory in the digital labor field. According to Self-Determination Theory, individuals can be proactive and engaged in their work when the social conditions they are situated in are autonomy-supportive. Conversely, individuals can be passive and alienated in their work when they experience social conditions as externally controlled. Under algorithmic control and given individuals' dual cognitive appraisal of stress, this study confirms the double-edged sword effect of algorithmic stress. By simultaneously integrating the opposing yet coexisting dual mechanisms of “job crafting” (proactive need satisfaction path) and “relative deprivation” (reactive need frustration path), this study constructs a relatively comprehensive and dialectical theoretical model. This not only reveals the complexity of algorithmic stress outcomes but also enriches the explanatory boundary of Self-Determination Theory regarding “how the external environment shapes motivation and behavior by influencing psychological need satisfaction,” particularly in the context of non-standard work under high technical control.

### 5.3 Practical implications

Platforms' focus should shift from a “control logic” to an “empowerment logic,” and pay more attention to workers' psychological needs into algorithm design. The research results indicate that relying solely on algorithms for accurate control exacerbates workers' sense of relative deprivation and undermines their long-term career commitment. Therefore, platforms should optimize algorithmic management. The main pathway is to make platform algorithms transparent.

First, platforms should enhance the knowability of task assignment rules, including transparent time calculation, visualized route planning, and public disclosure of order dispatch logic. This allows riders to know in advance the dynamic weight coefficients (e.g., weather coefficient, traffic condition coefficient) for order assignment and adjust their order acceptance strategies accordingly.

Second, platforms should develop an “intelligent dispatch algorithm based on gig workers' autonomous preferences,” such as allowing riders to preset their “preferred pickup areas,” “desired working time slots,” and “maximum number of concurrent orders,” based on which the system intelligently dispatches orders.

Third, platforms can introduce a “cross-platform order acceptance guarantee” policy to further enhance gig workers' time autonomy. This policy would allow gig workers to accept orders from other platforms without compromising the order quality on their primary platform, thereby increasing their labor income.

Forth, platforms should improve the operational feasibility of gig workers' rights protection, including clear basis for penalties, transparent income mechanisms, and well-defined data ownership. They can establish algorithm advisory groups that allow gig workers to directly participate in the deliberation of algorithmic rules. At the same time, they should strengthen social communication and submit to public supervision. Platforms should collect and address supervisory and critical feedback through measures such as user surveys, public and media open days, dedicated public consultation sections, and algorithmic advisory hotlines.

### 5.4 Limitations and future directions

This study has several limitations that warrant attention in future research: First, This study collected data only in China to explore the relationship between algorithmic stress and gig workers' career commitment. Future research could consider cross-national comparisons to enhance the generalizability of the conclusions. Second, this study treated algorithmic stress as a unitary construct for measurement and testing. While this helps grasp its overall effect macroscopically, it overlooks the potentially differentiated influence mechanisms of its various dimensions (e.g., regulation stress, time stress, alienation stress) on career commitment. Future research could conduct more fine-grained analyses. Third, this study focused on mediating mechanisms, revealing two competing pathways (job crafting and relative deprivation). However, it did not consider potential moderating effects. Future research could explore boundary conditions such as individual traits (e.g., psychological capital—resilience, optimism, self-efficacy; proactive personality; algorithmic literacy; or traditional career values) and work context factors (e.g., social support, income level and stability) to construct a more comprehensive and contextualized theoretical framework, thereby deepening our understanding of the complex relationship between algorithmic stress and gig workers' career commitment. Forth, this study includes all working-age gig workers in the sample. However, given that gig platforms are increasingly attracting younger workers (especially Generation Z), we believe it is necessary to specifically discuss the issue of young gig workers. Although young people have high digital literacy and a preference for flexible employment, they tend to have relatively limited work experience and weaker risk resilience, which may make them more sensitive to algorithmic stress. For example, algorithmic real-time ranking and negative review mechanisms may exacerbate young gig workers' income anxiety and job insecurity. At the same time, young workers may also be more inclined to view algorithmic challenges as growth opportunities, thereby exhibiting different coping strategies. Therefore, future research could further examine the moderating role of age and explore differentiated support strategies for young gig workers to promote the sustainable development of the gig economy.

## Data Availability

The raw data supporting the conclusions of this article will be made available by the author, without undue reservation.
